# Patient and primary care practitioners’ perspectives on consultations for fibromyalgia: a qualitative evidence synthesis

**DOI:** 10.1017/S1463423623000506

**Published:** 2023-09-26

**Authors:** Ailish Byrne, Katherine Jones, Michael Backhouse, Fiona Rose, Emma Moatt, Christina van der Feltz-Cornelis

**Affiliations:** 1 York Trials Unit, Department of Health Sciences, University of York, Heslington, York, UK; 2 Warwick Clinical Trials Unit, University of Warwick, Coventry, UK; 3 Mental Health and Addictions Research Group, Department of Health Sciences, Hull York Medical School, University of York, Heslington, York, UK; 4 Institute of Health Informatics, University College London, London, UK

**Keywords:** fibromyalgia, GP perspectives, patient perspectives, primary care consultations, qualitative evidence synthesis

## Abstract

**Background:**

Fibromyalgia presents a challenge to both the patients experiencing symptoms and the staff aiming to treat them. This qualitative review aimed to synthesise how patients and practitioners experience primary care consultations, develop a rounded picture of how they perceive each other, the challenges to primary care consultation and how they might be tackled.

**Methods:**

CINAHL, Embase, CENTRAL and Medline were searched from inception to November 2021. Qualitative studies were included if they explored the perspectives and experiences of either fibromyalgia patients or primary care practitioners. Quantitative data, studies not published in English, not set in primary care or that did not distinguish the type of patient or clinician were excluded. Included studies were analysed using thematic synthesis and their quality assessed.

**Results:**

In total, 30 studies met the inclusion criteria. Thematic synthesis identified three overarching themes: (1) life turned upside down – exploring the chaos experienced by patients as they seek help; (2) negative cycle – highlighting how patient and practitioner factors can create a detrimental cycle; and (3) breaking the cycle – validating patient–doctor relationships underpinned by clear communication can help break the negative cycle.

**Conclusions:**

Fibromyalgia patients experience uncertainty and chaos that can clash with the attitudes of GPs and the help they can feasibly provide. Difficult consultations in which neither the GP nor patient are satisfied can easily occur. Promoting supportive, reciprocal and open patient–doctor relationships is essential. Future research is required to further explore GP attitudes and to develop an intervention that could improve consultations, patient outcomes and GP satisfaction.

## Background

Fibromyalgia (FM) is characterised by widespread pain, fatigue and cognitive dysfunction (Doebl *et al*., 2021). FM has an estimated prevalence of 5.4% within the United Kingdom (UK) (Jones *et al*., 2015) and places significant burden on patients, primary care and the wider National Health Service (NHS) (Hughes *et al*., 2006; Soni *et al*., 2020).

FM is challenging in part due to the range of symptoms. These symptoms overlap with numerous other conditions, such as chronic fatigue syndrome (Åsbring and Närvänen, 2003) and functional neurological disorder (FND) (van der Feltz-Cornelis *et al*., 2021). There is evidence of a correlation between FM and psychiatric disorders, with 13–80% of FM patients having comorbid anxiety or depression (Galvez-Sánchez *et al*., 2019). Scepticism towards FM patients can be driven by invisible symptoms (e.g., pain) and the stigma of FM being a ‘womens’ illness’ (Sallinen *et al*., 2019). There are often inconsistencies between General Practitioners (GP) in diagnosis and treatment (Arnold *et al*., 2016), and there is currently no objective diagnostic test to confirm FM. FM patients often undergo numerous medical tests that come back normal (Goldenberg, 2009), are frequently misdiagnosed or have a delay receiving a diagnosis (Baron *et al*., 2014). Conflicting explanations for FM persist. For example, whilst FM is classified under chronic primary pain within the International Classification of Diseases (ICD-11 for Mortality and Morbidity Statistics, 2022), it is also classified as a ‘medically unexplained symptom’ condition – in which thoughts, feelings and stressors may explain physical symptoms – by the Royal College of Psychiatrists (RCPSYCH, 2023). A UK-based survey reported that whilst 66% of GPs felt they could do more to help FM patients, 41% felt that the criteria for FM is unclear and 30% were uncertain about treatments (Hayes *et al*., 2010). Over 1 in 5 (23%) stated that FM patients are ‘malingerers’ and 76% found that FM patients are time consuming and frustrating (Hayes *et al*., 2010).

Research with FM patients describes a long journey to validation, a diagnosis often taking years to obtain, featuring interactions with a ‘merry-go-round’ of clinicians – some of which questioned the legitimacy of their illness (Mengshoel *et al*., 2018). Whilst patients emphasise the importance of trust and the patient–doctor relationship to their prognosis, 38% delay visiting their doctor because they fear not being taken seriously, and 59% report difficulties trying to communicate their symptoms (Choy *et al*., 2010; Doebl *et al*., 2020).

Despite these difficulties, there are recommendations that FM should be managed within primary care as GPs are patient’s first point of contact, particularly for early intervention (Endresen, 2007; Rheumatology GIRFT Programme National Speciality Report, 2021). In which case, there is an urgent need to improve the experience of consultations for both FM patients and GPs.

This review aimed to explore the perspectives and experiences of FM patients and primary health-care clinicians. It is hoped that by synthesising the evidence from both patients and clinicians, it will create a holistic view of what issues are experienced and how they may be overcome.

## Methods

This qualitative evidence synthesis was prospectively registered on PROSPERO (registration record CRD42020201717) and followed the Cochrane Handbook for Qualitative Evidence Synthesis guidelines (Noyes *et al*., 2022). Reporting has been guided by the ENTREQ checklist (Tong *et al*., 2012).

The criteria provided in Table 1, developed using the SPIDER framework (Cooke *et al*., 2012), were used to assess potentially eligible studies.

**Table 1.** Inclusion and exclusion criteria (SPIDER)

	Inclusion criteria	Exclusion criteria
Sample	1.) Patients (adults, 16+) who have been given a diagnosis of fibromyalgia after having sought and experienced one or multiple health-care consultations. 2.) Clinicians who are registered health-care practitioners in the fields of General Practice, Rheumatology, Physiotherapy (etc.), who have undertaken consultation in primary care with patients presenting with Fibromyalgia symptoms.* * This criteria was amended in January 2022 to exclude secondary or tertiary practitioners and to only include health care practitioners in the field of General Practice (GPs or nurses).	Patients not diagnosed with FM or patients under the age of 16. Clinicians other than primary care General Practitioners or nurses.
Phenomenon of interest	The perspectives and experiences of both patients diagnosed with fibromyalgia and of the primary health-care clinicians who conduct consultations, including (but not limited to); perceived barriers and facilitators to diagnosis, experiences of providing and receiving treatment and the perceived and described attitudes of each party.	Studies that did not make it explicit that the data referred to a fibromyalgia patient, GP or primary care nurse. E.g., statements or quotes that referred to ‘doctor’ or ‘physician’ without indicating their speciality. E.g: studies that included MNYES patients without specifying FM, or papers that used a mixed population and did not state which quotes matched to which patient.
Design	Qualitative and mixed methods studies. Interviews, focus groups, open surveys or other established qualitative methods. Studies must have used established qualitative analytical approaches (e.g., thematic analysis) to analyse the findings. Publications from all countries were suitable for inclusion.	Quantitative methods studies or papers that were not full text (e.g., abstracts, protocols or posters). Studies had to be published in English or with an English translation.
Evaluation	Experiences, attitudes, behaviours, barriers and facilitators to primary care consultation for fibromyalgia.	
Research type	Qualitative evidence synthesis	

### Data sources

A pre-planned search strategy for four databases (MEDLINE, Embase, CENTRAL and CINAHL) was developed through collaboration between three authors (AB, MB and CVDFC). The search strategy combined key terms for the populations and phenomenon of interest with terminology for qualitative methodology using the Boolean operator AND (Appendix 1). Publications from any country were eligible if they were in English or with an English translation. Databases were searched from inception to November 2020, with an updated search between November 2020 and 2021. Manual searches of reference and citation lists were completed for studies meeting the inclusion criteria.

### Screening

Titles were screened by AB before abstract and full-texts were screened independently by two authors (AB and KJ/MB). The systematic review software Rayyan was used for screening and the blinded recording of decisions. Discrepancies were resolved by a third author (CVDFC). An inclusion/exclusion checklist was used to promote consistency.

A data extraction form was piloted by two authors (AB and MB). The Google form was designed to capture key characteristics of the included papers and participant characteristics (e.g., geographical location, study population and method of data collection). Data extraction was conducted by two authors independently (AB and MB/FR/EM).

### Literature synthesis

PDF versions of all the included studies were downloaded for manual data extraction and synthesis. Coding was line-by-line and inductive, using quotes and interpretations provided within the ‘results’ and ‘discussion’ sections. Themes given by the authors were not coded and compared as the study aimed to provide a synthesis across patient and clinician data, rather than to review the consistency of themes across studies, and as we anticipated the inclusion of mixed-population studies. However, familiarisation with the included studies and wider literature may have influenced coding. Initial coding frameworks for patient and clinician data were developed by two authors (AB and KJ) independently coding three papers and reaching a consensus on initial second-order constructs. Thematic synthesis was used for analysis as a method useful for assessing intervention need, whilst its inclusion of line-by-line coding allows for the translation of concepts across studies (Thomas and Harden, 2008; Barnett-Page and Thomas, 2009). Coding frameworks were referred to throughout and expanded upon based on the emerging data. The final frameworks mapped out the translation of data from relevant first- and second-order constructs into codes, sub-themes and themes.

The coded data were re-read alongside the study aims to assist in the development of themes. Themes were considered as written interpretations, mapped onto data and visualised using diagrams. Twenty-nine codes were mapped onto three overarching themes by AB. Once themes had been developed, all authors reviewed the data coding, mapping and interpretations. AB and KJ were mindful throughout analysis of their biases as patients who have experienced difficult GP interactions and, in the case of AB, having family with FM. Discussions were had during analysis which allowed for biases to be acknowledged and checked. On one occasion was this notable enough to warrant discussion, after which a consensus was reached.

Themes were discussed with a patient advisor (JB, symptomatic since 2013, diagnosed with FM in 2015). JB reviewed the themes, providing detailed feedback to each and how they related to their experience. Themes were felt to resonate to their experience but were refined according to their feedback.

### Quality Assessment (QA)

Quality assessment (QA) was conducted by two authors independently (AB and KJ/FR/EM) using the Critical Appraisal Skills Programme (Critical Appraisal Skills Programme, 2018) checklist. Discrepancies were resolved through discussion.

## Results

### Study characteristics

**Figure 1.**
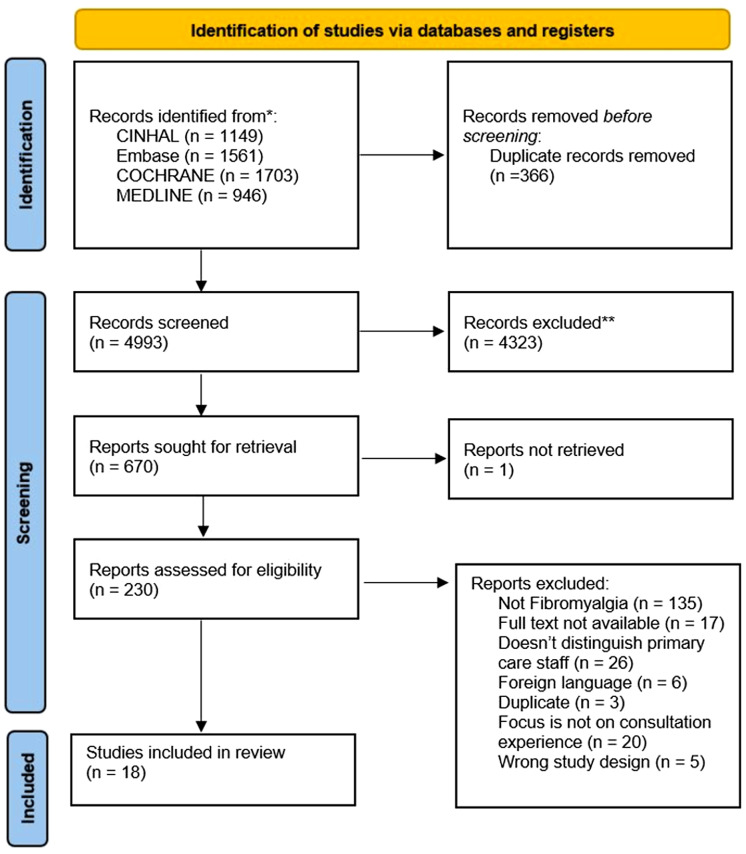
PRISMA flow diagram (Page *et al*., 2021). Initial search (inception –November 2020).

**Figure 2.**
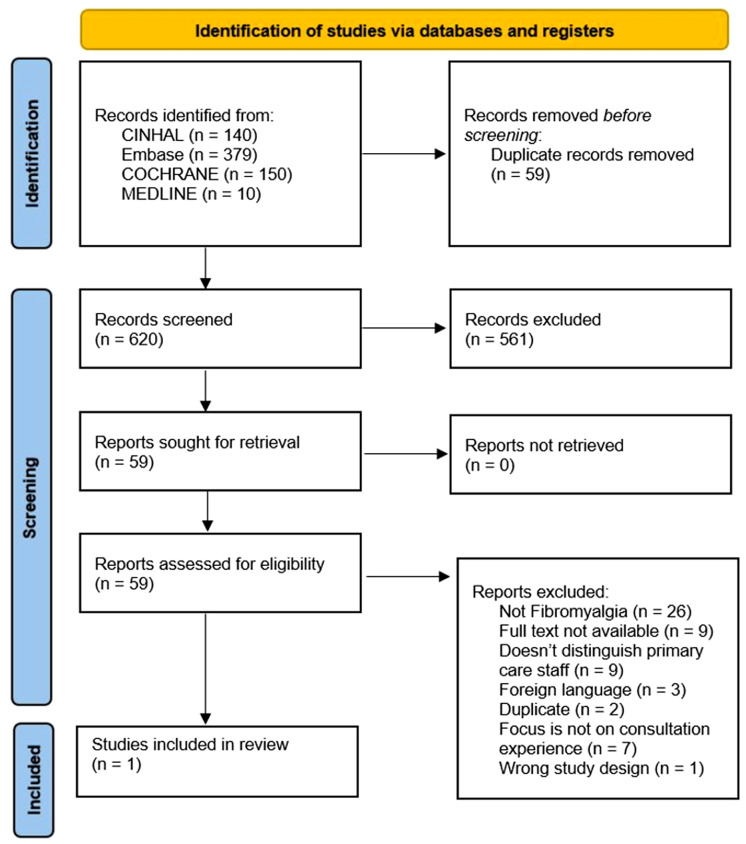
PRISMA flow diagram (Page *et al*., 2021). Second search (November 2020–2021).

The search strategy returned 6038 studies. Altogether, 289 were full text screened, and 19 studies met the inclusion criteria (Figures 1 and 2). Reference and citation lists revealed another 11 papers, leading to 30 included papers.

The characteristics and codes from the 30 studies are illustrated in Tables 2 and 3 and further in Appendix 2. Two papers appear to include different data from the same study (Madden and Sim, 2006, 2016), indicated with * within Table 3.

**Table 2.** Characteristics and codes from clinician studies.

Author	Country	Year	Aim	Participants	Qualitative methodology	Data analysis	Themes
Briones-Vozmediano *et al*.	Spain	2017	To explore health service providers perceptions regarding fibromyalgia patients in Spain and to analyse possible consequences of these perceptions	12 health professionals Included 1 GP	Semi structured interviews	Content analysis	- GP’s attitudes towards patients (doubt, scepticism, unkind) - Typecasting/judgements of FM (condition and patients)
Wainwright *et al*.	UK	2006	Not clear	14 healthcare workers	Interviews	Thematic analysis	- Lack of knowledge - Perceived importance of diagnosis - Typecasting/judgements of FM (condition and patients) - FM is purely psychological - FM is not real - GP shopping
Britten *et al*.	UK	2000	To identify misunderstandings between patients and doctors that have potential or actual adverse consequences for taking medicines	20 GPs and 35 participants (1 FM)	Semi-structured interviews.	Not given	- Typecasting/judgements of FM (condition and patients) - Lack of communication
Hayes *et al*.	Canada	2010	To gain a better understanding of the nature of the patient–provider relationship in fibromyalgia and the underlying knowledge and attitudinal challenges	189 GPs, 139 specialists, two nurses and 18 patients. 39 individuals from this sample partook in the qualitative work.	Discussion groups and semi-structured interviews	Grounded theory analysis	- FM is not real - Lack of knowledge - Lack of effective treatments - GP’s attitudes towards patients (doubt, scepticism, unkind) - Typecasting/judgements of FM (condition and patients) - FM isn’t real - GP unable to help - Paternalistic model - No one wants to look at it
Hellström *et al*.	Sweden	1998	To trace out the meaning structure that guides GPs’ and rheumatologist experiences of and attitudes towards FM and FM patients	10 rheumatologists and 10 GPs	Interviews.	Not given	- Perceived importance of diagnosis - GPs unable to help - Strained patient–doctor relationships
Briones-Vozmediano *et al*.	Spain	2013	To explore specific areas of the health care process that professionals and patients may consider unsatisfactory	9 health professionals and 12 FM patients Included 1 GP	Semi structured interviews	Discourse analysis	- GPs unable to help - Strained patient–doctor relationships

FM = Fibromyalgia, GP = General Practitioner.

**Table 3.** Characteristics and themes from FM patients

Author	Country	Year	Aim	Participants	Qualitative methodology	Data analysis	Themes
Chen and Swaminathan	USA	2020	To examine, from patients perspectives, factors that influence the formation of effective patient–provider relationships	23 FM patients	Semi-structured 1-1 interviews	Interpretative phenomenological analysis	- Perceived importance of diagnosis
Cooper and Gilbert	South Africa	2017	To bring insight into the particular navigating tools utilised by people living with medically ill-defined conditions in attaining a diagnosis of fibromyalgia in SA	15 FM patients	Semi structured interviews	Thematic analysis	- Positive GP behaviours (listening, validating, supportive, understanding, proactive) - Patients as self- advocates - Impact of interactions
Diver	UK	2012	To capture the lived experiences of individuals newly diagnosed with FMS and explore how they change over time	23 FM patients	Semi-structured interviews	Thematic analysis	- Biomedical model over subjective experience of symptoms - Lack of effective treatments - Lack of confidence in GP - Perceived importance of diagnosis - GP’s attitudes towards patients (doubt, scepticism, unkind) - Lack of communication - GP as ‘gatekeepers’/an information source - Patients as self-advocates - Societal expectations of patients
Durif-Bruckert, Roux and Rousset	France	2015	To understand the medication experience of patients with fibromyalgia and their relationship with the doctors	35 FM patients	Interviews	Content analysis	- Lack of effective treatments - GP’s attitudes towards patients (doubt, scepticism, unkind) - Positive GP behaviours (listening, validating, supportive, understanding, proactive)
Paulson, Norberg and Danielson	Sweden	2002	To describe how men living with fibromyalgia-type pain experienced being patients in the Swedish health care system.	14 FM patients	Narrative interviews	Content analysis	- Lack of continuity of care - Lack of time - GP’s attitudes towards patients (doubt, scepticism, unkind) - Lack of communication
Reibel and Pearson	USA	2017	To gain an understanding of the lived experiences of women with fibromyalgia	3 FM patients	Phenomenological interviews	Thematic analysis	- Lack of effective treatments - Perceived importance of diagnosis - Lack of communication
Rodham, Rance and Blake	UK	2010	To extend previous research by investigating the lived experiences both of those with FMS and their spousal carers.	4 FM patients and their partners	Interviews	Phenomenological analysis	- GP’s attitudes towards patients (doubt, scepticism, unkind)
Boulton	Canada & UK	2019	To examine the diagnostic process and to understand how people with FM feel about this label	31 FM patients	Interviews	Narrative analysis	- Exhaustive process - Perceived importance of diagnosis
Undeland and Malterud	Norway	2007	To explore experiences and consequences of the process of being diagnosed with fibromyalgia	11 FM patients	Interviews	Systematic text condensation	- GP’s attitudes towards patients (doubt, scepticism, unkind) - Patients understanding of FM
Wuytack and Miller	Belgium	2011	To gain a better understanding of the subjective experience of fibromyalgia, focusing on the personal, occupational and social impact of the condition on patients’ lives	6 FM patients	Structured interviews	Thematic framework analysis	- GP’s attitudes towards patients (doubt, scepticism, unkind)
Lempp, Hatch, Carville and Choy	UK	2009	To understand the experience of living with FM, using qualitative interviews.	21 FM patients	Semi-structured interviews	Content/discourse analysis	- Lack of time - Positive GP behaviours (listening, validating, supportive, understanding, pro-active)
Briones-Vozmediano *et al*.	Spain	2013	To explore specific areas of the health care process that professionals and patients may consider unsatisfactory	9 health professionals and 12 FM patients	Semi structured interviews	Discourse analysis	- GP unable to help - Strained patient–doctor relationships
Muraleetharan *et al*.	USA	2018	To understand FM by examining multiple impacts of fibromyalgia on men in regard to interactions in society and the U.S. health system	1163 (805 with FM) patients	Qualitative survey	Thematic analysis	- Lack of communication - Positive GP behaviours (listening, validating, supportive, understanding, proactive) - Societal expectations of patients
Gjengedal *et al*.	Norway	2019	To explore what persons who have been critically ill and persons suffering from long-lasting chronic muscle pain, consider as important research topics	23 patients (14 with FM)	Focus groups and phenomenological reflections	Phenomenological reflections	- Perceived importance of diagnosis
Britten *et al*.	UK	2000	To identify misunderstandings between patients and doctors that have potential or actual adverse consequences for taking medicines	20 GPs and 35 participants (1 FM)	Semi-structured interviews.	Not given	- Typecasting/judgments about FM (condition and patient) - Lack of communication
Hayes *et al*.	Canada	2010	To gain a better understanding of the nature of the patient–provider relationship in fibromyalgia and the underlying knowledge and attitudinal challenges	189 GPs, 139 specialists, two nurses and 18 patients	Discussion groups and semi-structured interviews	Grounded theory analysis	- Lack of effective treatments
Thorne *et al*.	British Columbia	2004	To portray how individuals with fibromyalgia describe interactions with health care providers and to highlight patterns of communication that were perceived as helpful or unhelpful.	11 FM patients	Interviews	Constant comparative analysis	- Positive GP behaviours (listening, validating, supportive, understanding, proactive)
Armentor	USA	2017	To explore the various approaches used by individuals with FM to manage their social roles and negotiate their relationships with others	20 women with FM	Semi-structured interviews	Grounded theory analysis	- GP’s attitudes towards patients (doubt, scepticism, unkind) - FM is purely psychological
Agyare	UK	2020	To uncover the ways in which patients diagnosed with fibromyalgia talked about their experience of fibromyalgia.	7 women with FM	Semi structured interviews	Foucauldian discourse analysis.	- Lack of continuity of care - Lack of knowledge - Lack of effective treatments - GP’s attitudes towards patients (doubt, scepticism, unkind) - FM is purely psychological - FM isn’t real - Patients as self-advocates - ‘GP shopping’ - GPs unable to help
Diviney and Dowling	Ireland	2015	To explore the lived experience of people diagnosed with fibromyalgia	9 FM patients	Interviews	Lifeworld approach	- Lack of knowledge
Egeli *et al*.	Canada	2008	To examine patients’ descriptions of both positive and negative physician encounters; to provide patient recommendations for improved care and consider the implications of these findings for nursing practice.	42 FM patients	Open- ended, qualitative questionnaire	Descriptive approach	- Patients as self-advocates
Hiort, Lindau and Löfgren	Sweden	2017	The purpose was to explore interview data from young adults with long-standing pain about their experience of contacts with caregivers in a primary care setting	Young people (20 – 31 years) with long term pain, including 4 with fibromyalgia	Interviews	Thematic content analysis	- Lack of communication
Chen	USA	2015	To explore the illness journeys of fibromyalgia patients	23 FM patients	Semi-structured interviews	Thematic analysis,	- Patients as self-advocates - ‘GP shopping’
Abokdeer	UK	2019	To capture service users’ and service providers’ views and experiences of fibromyalgia and its management, including their views on the journey to diagnosis, current practice availability, emotional and life experience, information seeking and experiences of seeking and receiving health care	12 women with FM	Semi-structured interviews	Framework analysis	- Lack of continuity of care - Lack of knowledge - Patients physical appearance not matching their internal experience - Lack of confidence in GP - FM is not real - Positive GP behaviours (listening, validating, supportive, understanding, proactive) - Impact of interactions - Lack of practical information from GP
Madden and Sim*	UK	2006	To explore the creation of meaning in a medically unexplained disorder, fibromyalgia syndrome (FMS)	17 FM patients	Semi-structured interviews	Induction-abduction method and documentary analysis	- Lack of communication
Skop	Canada	2015	To explore the retrospective and ongoing healthcare experiences of men and women who have a diagnosis of fibromyalgia.	35 FM patients	Focus groups and interviews	Grounded theory	- Lack of effective treatments - GP’s attitudes towards patients (doubt, scepticism, unkind) - Positive GP behaviours (listening, validating, supportive, understanding, pro-active) - Collaborative relationship - Impact of interactions - Strained patient–doctor relationships - Paternalistic model
Madden and Sim*	UK	2016	To explore the experience of diagnosis in FMS and to evaluate the contribution that the negotiated order, viewed alongside other perspectives, can make to an understanding of this experience.	17 FM patients	Semi-structured interviews	Unclear	- Biomedical model over subjective experience of symptoms - Societal expectations of GP - GP’s attitudes towards patients (doubt, scepticism, unkind) - Patients as self-advocates

* Associated papers.

FM = Fibromyalgia, GP = General Practitioner.

Six papers explored how primary care practitioners experience consultations for FM. All six studies included mixed healthcare populations (e.g., GPs and secondary/tertiary care doctors). None recruited primary care nurses. Totally, 28 studies explored FM patients’ experiences, with the majority exclusively including FM as their patient population. Studies were conducted across ten countries, predominantly used qualitative interviews, and reported a range of analyses (Tables 2 and 3).

### Quality assessment

The studies ranged in quality. Whilst 7/30 were of high quality, 23 displayed at least one methodological limitation (Appendix 3). Limitations most commonly related to the relationship between the researcher and participant, as 22/30 papers reported this insufficiently. Other common limitations surrounded the rigour of data analysis (7/30), data collection (6/30), statement of study findings (6/30) and ethics (5/30).

### Thematic synthesis

Three overarching themes were identified: life turned upside down, negative cycle and breaking the cycle.

#### Theme 1: Life turned upside down

This theme lends itself to sociological theories of chronic illness, including biographical disruption and legitimation (Bury, 1982, 1991) and chaos narratives (Nettleton *et al*. 2005) by reflecting how once FM symptoms start patients may find themselves no longer meeting their own expectations and those of society (Supplementary material 2).

Western societal expectations dictate that if you feel sick you go to your GP (Diver, 2012, p.192). The GP will identify the problem, offer treatment and resolve your illness. FM patients usually begin their journey by experiencing invisible symptoms that stop them from doing usual activities and actions that they previously could and would expect to be able to, do. Following the expected pathway, they seek help from their GP. Contrary to normal expectations, the studies highlighted how GPs were often unable to help their symptoms, identify what was causing their illness and sometimes did not believe their illness was real.

‘They considered themselves to be fulfilling social and cultural obligations by attending their GP and being compliant with tests and treatments offered and yet their situation remained the same or worsened’. Author quote. Diver (2012). p.224.

Patients often reported receiving alternate explanations for their illness that were later proved wrong. These explanations did not fully fit their symptom experience, but patients were willing to accept them due to the trust they placed in their GP.

‘You go blindly on, you’re not really satisfied, but then I thought well there’s people a lot worse off than me. If that’s what she [GP] says it is, that’s what it must be’. Patient quote. Madden and Sim (2016). p.97.

Some patients found the consultation itself flipped upside down – from an interaction designed to help them to an interrogation about their credibility. Patients were questioned on the legitimacy of their illness, dismissed and encountered unsympathetic GPs. Patients’ credibility was an issue both prior and post-diagnosis.

‘They still didn’t want to believe it…I had to change GP. I err went through a very bad time with the GP, who just said I didn’t have anything wrong with me, still wouldn’t, you know, didn’t want to accept the diagnosis’. Patient quote. Rodham *et al*. (2010). p.71.

The studies illustrated that some patients felt their credibility in question because they experienced invisible symptoms, received inconclusive diagnostic tests and because their physical experience often did not match their physical appearance.

‘Participants described how they were questioned at times about whether they genuinely had been ill and suffering with pain by people at work, and even by GPs, as the result of looking physically well’. Author quote. Abokdeer (2019). p. 89.

Some patients reported experiencing scepticism, dismissal and no objective confirmation of their illness or its severity from their GP, despite pursuing the socially correct path to validation. This led patients to feel isolated, uncertain and to begin doubting themselves.

‘They were influenced by their inability to live their “normal” lives and began to lose confidence in their own explanations for their symptoms as well as those provided by their GP’. Author quote. Diver (2012). p.196.

In line with Nettleton *et al*. (2005), across studies it appeared to be the uncertainty that was driving the ‘chaos’ patients felt. Diver (2012, p.223) reported that patients who received a diagnosis early experienced less ‘chaos’. This may be because diagnosis provided patients with a certainty in a period of extreme uncertainty – allowing them insight into the severity of their condition, the reality of what their illness meant for their lives, and a degree of legitimation (Bury, 1991).

For patients filled with uncertainty and chaos, it led to actions such as ‘GP shopping’ – consulting multiple GPs – and self-advocacy. This dislike of uncertainty appears to clash with GP narratives, in which they appeared comfortable sitting in the uncertainty with the option to refer the patient on.

‘[…] the interviews gave the impression that the GPs were more accustomed to living with uncertainty and with a less structured practice than hospital clinicians’. Author quote. Hellström *et al*. (1998). p.235.

The traditional paternalistic model assumes that the doctor knows all – including what your illness is, what decisions, actions and treatments should be given – and that an ‘unwavering trust’ should be given (Lee, 2007; Murgic *et al*., 2015). We theorise that the expectation this model has set up presents a conflict for FM patients if their GP doesn’t have the knowledge and/or options to help – further driving the feeling that their lives had been turned upside down.

‘It’s very stressful because when you go to the GP I think you want them to know. You expect them to be able to pinpoint at what you’re suffering from’. Patient quote. Agyare (2020). p.114.

Moreover, despite a diagnosis potentially reducing the experience of chaos there was a sense that patients were forced to assimilate their status as a FM patient into their identity and were disappointed with what that meant.

‘She was disappointed, because she knew the word ‘fibromyalgia’ would cling to her, even if she died of cancer or a heart attack’. Author quote. Undeland and Malterud (2007). p.252.

#### Theme 2: Negative cycle

Across the included studies, there was evidence of positive and negative interactions. Within the negative narratives, there emerged evidence of a cyclic relationship in which GP and patient factors fed into each other, worsening the consultation process over time (Supplementary material 3).

Lack of knowledge was predominant within this theme from both perspectives and fed into all aspects – from beliefs about the underlying mechanisms of FM and its existence, the knowledge held by GPs and patients, to the limited treatment options available.

‘It does have some criteria, and its complex, and we definitely don’t understand it as well as we need to’. GP quote. Hayes *et al*. (2010). p.387.

The challenges created by the lack of knowledge were further exacerbated by negative GP attitudes, including the belief that FM patients are ‘soft’ and need to ‘liven up’ (Briones-Vozmediano *et al*., 2018, p.1682). Doubt over the validity of FM as a disease was also exhibited, particularly over the underlying cause (biomechanical or psychosocial).

‘They are patients that…towards them whom I feel rejection, I have to admit’. GP quote. Briones-Vozmediano *et al*. (2018). p.1682.

‘I’m not convinced […] That’s my problem with Fibromyalgia, I’m not convinced at all’. GP quote. Hayes *et al*. (2010). p.387.

Further difficulty and doubt arose due to a lack of objective markers of the disease, with patients frequently returning inconclusive medical tests and being unable to confirm their symptoms objectively.

‘I kept going back to the GP over and over again with the pain and he sent me for various tests but couldn’t find anything wrong’. Patient quote. Boulton (2019). p.812.

Patient narratives demonstrated an expectation that the GP should have all the answers to their illness. GPs’ were viewed as gatekeepers to treatments and information. This is likely to be further driven by patients’ dependence on their doctor confirming their sickness to other authorities, for example; to claim benefits or receive sick leave. Conflict arose when patients did not receive the help they expected, gained no knowledge about their illness or were offered diagnoses that did not fit.

‘The GP, you know, didn’t have a clue about fibromyalgia, didn’t have a clue about what my pain was’. Patient quote. Abokdeer (2019). p.81.

Whilst patients felt that they were fulfilling their expected role, they were faced with conflict when the GP did not appear to fulfil theirs. In an attempt to resolve this conflict, patients learnt to self-advocate, seeking out other GPs and asking for further tests, treatments and referrals.

‘Participants talked about experiencing resistance towards their doctors and GPs and talked about trying to convince them that they were ill, hence the multiple visits, long diagnostic journeys and insistence on referrals to other medical specialists’. Author quote. Agyare (2020). p.102.

This self-advocacy clashes with the paternalistic model, in which patients are expected to take a passive role (Bissell *et al*., 2004). Instead, it pushes a collaborative, patient-centred agenda in consultations where the GP may not always be willing to accept this model. GP narratives, in turn, illustrated a resistance towards patients and the development of an understanding that these are a difficult patient group, whose use of healthcare is reflective of attention-seeking behaviour. Concern was expressed that they could ‘fall in’ with patients’ agendas (Hayes *et al*., 2010. p.389.). There was a sense that GPs began to typecast patients, wanting a test to assess how ‘worth it’ patients were to work with (Hayes *et al*., 2010. p.389) and consciously placing limits on what they were happy to sign off on.

‘They feel mentally like no one understands them, and this is part I think, of the characteristics of the profile of fibromyalgia…that wherever they go no one pays attention’. GP quote. Briones-Vozmediano *et al*. (2018). p.1682.

The cycle rounds off with recognition of the limited treatment options. There is no cure for FM and the limited number of treatment options available are not always effective. Moreover, when prescribing pain medication, the available drugs often have significant side-effects and can cause dependency, leading GPs to be justifiably cautious. GPs are likely to feel a degree of helplessness and frustration when consulting with FM patients because they have limited – possibly ineffective – options.

‘No matter what you give them, the pain doesn’t go away’. GP quote. Briones-Vozmediano *et al*. (2013). p.21.

Evidence is emerging that the knowledge, attitudes, actions and options of GPs and patients can negatively feed into each other, interacting with medical models to produce a detrimental cycle that effects both parties. The cycle teaches patients that their GP is nolt interested in their symptoms, and as a result they may withhold information, experience isolation and a worsening of their symptoms. GPs learn that FM patients are unrewarding, difficult, frustrating and perceive that they will never be able to do enough to help. Factors within the cycle are likely to be exacerbated by practical barriers, including lack of time and continuity of care.

‘I think that the bottom line is no one really wants to look at it’. GP quote. Hayes *et al*. (2010). p.388.

#### Theme 3: Breaking the cycle

However, by no means was the only narrative chaos and conflict. Whilst the data were relatively thin and lacked data from the GP perspective, studies did provide evidence of supportive, facilitating patient–doctor relationships, with a clear goal to help patients (Supplementary material 4). In these narratives, neither the patient nor GP appeared to fall into the negative cycle illustrated in Theme 2.

Positive patient narratives described the importance of legitimisation, validation and being taken seriously. Legitimisation of FM led some patients to a better healthcare experience and outcomes.

‘She expressed that finding a GP who accepted her condition proved to be a crucial element in shaping her illness experience and outcomes positively’. Author quote. Cooper and Gilbert (2017). p.347.

Diagnosis appeared to be used by some GPs as a tool to provide patients with legitimacy, confirming that their condition was real whilst also allowing the GPs a natural transition to progress with their care plan. Diagnosis acted as a bridge between the GP and patient, allowing them both to meet a need. This is an interesting contrast to the narratives of those who appeared sceptical and were against providing the diagnosis, believing the label misleading, detrimental or of little value.

‘As well as reassuring the patient, the ascription of a diagnosis of Fibromyalgia may also mark a transition in the consultation, effectively bringing the “work up” to a close and leading to the deployment of treatment options’. Author quote. Wainwright *et al*. (2006). p.82.

Legitimisation was also facilitated through effective communication and reciprocal patient–doctor relationships. Patients who described their GP as engaging in active listening, being supportive and accepting and giving clear guidance were more satisfied and had better outcomes. One quote noted how the GP saying, ‘I understand how you feel’ provided the patient with a ‘sense of validation and legitimacy’ (Skop, 2015, p.180). This contrasts with negative accounts, in which GPs were described as ‘sympathy not there, empathy not there’ (Agyare, 2020, p.100), not providing ‘proper conversation’ (Paulson *et al*., 2002, p.91) and not giving ‘straight answers’ (Hiort *et al*., 2017, p.91). The need for clear, open and effective communication was consistently highlighted, as the studies described encounters with misaligned priorities, miscommunication and assumptions about the other party.

Medication presented an interesting example of miscommunication. Britten *et al*. (2000, p.486) described a FM patient wanting to stop their painkillers whilst their GP described the patient as someone who ‘loves taking medicines’. This disparity was evident in other narratives, with patients describing how they felt ‘fobbed off’ through their GPs use of medication, believing them to be an out used if they didn’t know what the problem was. ‘*They all prescribe different medication because they don’t really know what it is’. Patient quote. Hayes et al. (2010). p.388.*

Another overarching finding was the need to improve knowledge. Whilst GPs acknowledged FM as an uncertain condition that required better understanding, patients expressed that the overall lack of knowledge made it difficult for them to manage their FM, and that improvements would result in better patient outcomes.

‘If General Practitioners understood it yes, it would be a lot better for everybody and it would help I think more and more people with fibromyalgia to – not get better but of course feel better at least’. Patient quote. Diviney and Dowling (2015). p.5.

## Discussion

This review synthesised the experiences of GPs and FM patients from 30 qualitative studies published across 10 countries. The studies provide an in-depth understanding of how patients and GP’s experience primary care consultations for FM. The studies varied in quality, with 7 being assessed as high quality and 23 displaying at least one weakness. Findings from this review indicate that conflict between GPs and FM patients can exist within primary care, but that clear, effective communication and reciprocal patient–doctor relationships could help overcome these difficulties. A start would be to acknowledge the difficulties faced by each party. It needs to be acknowledged that when FM patients visit primary care, they are likely to be in a state of uncertainty and chaos. The experience of invalidation and inability to receive answers exacerbates this and can have a negative impact on their outcomes (e.g., anxiety/depression, Supplementary Material 1). GPs appeared to be more comfortable sitting in uncertainty than patients because they have the option to refer on. Considering the argument that GPs are best placed to manage FM (Endresen, 2007), the uncertainty may need to be addressed.

Whilst the NHS transitions into a more patient-centred model of healthcare, the authors speculate whether FM experiences greater resistance to this change than conditions with a recognised pathophysiology. Whilst the paternalistic model has constructed the doctor as a superior figure of knowledge, the transition to patient–doctor collaboration would require an admission that medicine knows very little about FM. GPs cannot have endless knowledge, and there should be an understanding from patients that they cannot know everything. Encouraging better communication and transparency about what GPs do know and can offer may ultimately improve both patient outcomes and GP satisfaction.

It is also important to consider these findings in relation to context. FM is controversial, having been considered psychogenic throughout the 19th–20th century (Pikoff, 2010), before being classified as a somatic symptom disorder by the Diagnostic and Statistical Manual of Mental Disorders (DSM-5) (Wolfe *et al*., 2014). FM is currently classified as a condition caused by stress and emotional distress by the RCPSYCH; however, there has been an increasing shift through the 21st century towards a physical explanation.

To guide clinicians, classification criteria for FM have been produced (Wolfe *et al*., 1990; Wolfe *et al*., 2010; Macfarlane *et al*., 2017). Whilst this review highlights that there is still much work required to improve consultations, as four out of six studies including GPs were conducted on or before 2010 perhaps their data simply does not reflect updated knowledge, criteria and the attitudinal shift seen over the last ten years. New guidance on the diagnosis of FM has been produced by the Royal College of Physicians (2022). It will be interesting to see how their implementation changes experiences going forward.

This review is supported by the wide breadth of studies included. Whilst the authors cannot state they achieved theoretical saturation as further studies could have provided novel information (Booth, 2016), the majority of data fit into existing codes. This review was developed following the Cochrane guidelines (Noyes *et al*., 2022) and ENTREQ checklist (Tong *et al*., 2012). Clear attempts to reduce bias were made through double abstract/full-text screening, data extraction, quality assessment and the development of coding frameworks. The review may be limited in that title screening and coding were done by a single author. Some relevant studies could have been missed, or different themes could have been derived if multiple authors had coded. However, we have mitigated against this due to the broad criteria applied at title screening, and as all authors – each with a range of perspectives and backgrounds (Appendix 4) – were required to check coding made logical sense to the data.

Another limitation was the inclusion of studies written in English or with an English translation. A more comprehensive search strategy would have included international databases, such as LILACS, improving the generalisability of this review and reducing the risk of language bias. However, as this review has framed the issue in the context of the UK NHS the inclusion of cross-cultural studies may limit the findings. As the NHS has a unique infrastructure, some data may not relate or be feasible. For example, ‘GP shopping’ may occur less within the NHS as there will be a limit to how many different GPs patients can see within their registered practice.

As AB and KJ identified personal experiences that could have biased interpretations, possibly causing patient narratives to be read more sympathetically, there is the potential for author bias. Efforts were made to minimise this through open conversation that allowed for biases to be checked.

Future research should include greater GP input, to understand if and how updated guidance has influenced modern practice and to explore which patient behaviours frustrate GPs and why. Research should also explore what causes a good consultation from the GP perspective – including if any patient actions could improve consultations. Moreover, this review cannot give an accurate estimate of how common these issues are. Considering the publication of later guidance, attitudes amongst GPs may have evolved since the survey by Hayes *et al*. (2010).

Research may wish to explore the implementation of an intervention to improve FM consultations within the NHS. Previous clinical trials have developed enhanced care interventions and have shown some initial, albeit limited, promise (Byrne *et al*. 2022). The MSS3 trial (Mooney *et al*. 2022) will soon provide data on the impact of symptom clinics within primary care, and future research may also wish to explore if other interventions could benefit patient outcomes and/or GP satisfaction within the context of the NHS.

## Conclusion

The findings from this review suggest that the chaos and uncertainty experienced by FM patients can clash with the attitudes and actions of GPs to produce a negative cycle that can undermine primary care consultations. Legitimisation, clear communication and effective patient–doctor relationships have been evidenced to break this cycle and may also improve patient outcomes and GP satisfaction. Future research should conduct further qualitative investigation into the GPs experience and focus on developing an enhanced care intervention that could be implemented within primary care to improve consultations for FM. However, to truly improve FM consultations the wider context needs to be considered, such as reaching a consensus between professions on the terminology, legitimacy, classification and likely aetiology of FM.

## Supplementary material

For supplementary material accompanying this paper visit http://doi.org/10.1017/S1463423623000506. click here to view supplementary material

## Appendix 1 Search strategy

1. Family practice
2. GP*
3. General Practitioner*
4. Primary care
5. Clinician*
6. Doctor*
7. Physician*
8. Health professional
9. Healthcare
10. Physio*
11. Rheum*
12. Medical specialist
13. First contact practitioners
14. First contact
15. First contact physiotherapist
16. Musculoskeletal
17. Pain clinic
18. 1 or 2 or 3 or 4 or 5 or 6 or 7 or 8 or 9 or 10 or 11 or 12 or 13 or 14 or 15 or 16 or 17
19. Fibromyalgia
20. Fibro
21. Chronic pain
22. 19 or 20 or 21 23.
23. Patient
24. Patient–doctor relationship
25. 23 or 24
26. Qualitative
27. Mixed Method
28. 26 or 27
29. Diagnosis
30. Treatment*
31. Therapy
32. Management
33. Confidence
34. Barrier*
35. Facilitator*
36. Experience*
37. Perspective*
38. Opinion*
39. 29 or 30 or 31 or 32 or 33 or 34 or 35 or 36 or 37 or 38
40. 18 or 25
41. 40 and 22
42. 41 and 39
43. 42 and 28

## Appendix 2 Further study characteristics

**Table:**

Title	Author	Year	Country	Aim	Participants	Sampling technique	Method of data collection	Method of data analysis	Type of publication
Factors in the building of effective patient–provider relationships in the context of Fibromyalgia	Chen and Swaminathan	2020	USA	To explore, from the patient’s perspective, key factors that influenced the formation of patient–provider relationships: information interactions, finding providers, and realising shared responsibilities.	23 FM patients	Convenience sampling via social media, previous survey respondents, support groups and university lists	Semi-structured 1-1 interviews	Interpretative phenomenological analysis	Paper
An exploratory study of the experience of fibromyalgia diagnosis in South Africa	Cooper and Gilbert	2017	South Africa	To bring insight into the particular navigating tools utilised by people living with medically ill-defined conditions in attaining a diagnosis of fibromyalgia in SA	15 FM patients	Purposive from informal peer networks	Semi structured interviews	Thematic analysis	Paper
Quest, chaos and restitution: A qualitative study of the experiences of individuals diagnosed with fibromyalgia syndrome	Diver	2012	UK	To capture the lived experiences of individuals newly diagnosed with FMS and explore how they change over time	23 FM patients	Theoretical sampling from a rheumatology clinic	Semi-structured interviews	Thematic analysis	Thesis
Medication and the patient–doctor relationship: a qualitative study with patients suffering from fibromyalgia	Durif-Bruckert, Roux and Rousset	2015	France	To understand the medication experience of patients with fibromyalgia and their relationship with the doctors derived from treatment negotiation.	35 FM patients	Convenience sampling from the rheumatology and internal medicine departments of a French University Hospital	Interviews	Content analysis	Paper
Men living with fibromyalgia-type pain: experiences as patients in the Swedish health care system	Paulson, Norberg and Danielson	2002	Sweden	To describe how men living with fibromyalgia-type pain experienced being patients in the Swedish health care system.	14 FM patients	Purposive sampling from a rheumatology hospital	Narrative interviews	Content analysis	Paper
Beyond the Pain: A Look into the Experiences of Women Living with Fibromyalgia	Reibel and Pearson	2017	USA	To gain an understanding of the lived experiences of women with fibromyalgia	3 FM patients	Purposive sampling from a FM support group	Phenomenological interviews	Thematic analysis	Paper
A qualitative exploration of carers’ and ‘patients’ experiences of fibromyalgia: one illness, different perspectives	Rodham, Rance and Blake	2010	UK	To extend previous research by investigating the lived experiences both of those with FMS and their spousal carers.	4 FM patients and their partners	Recruitment was done from a local FM support group	Interviews	Phenomenological analysis	Paper
Nothing and Everything: Fibromyalgia as a Diagnosis of Exclusion and Inclusion	Boulton	2019	Canada & UK	To examine the diagnostic process from the perspectives of people who have gone through it and to understand how people with FM feel about this label	31 FM patients	Recruitment through advertisements in FM support groups, public notice boards and referrals from other interviewees	Interviews	Narrative analysis	Paper
The fibromyalgia diagnosis: hardly helpful for the patients? A qualitative focus group study	Undeland and Malterud	2007	Norway	To explore experiences and consequences of the process of being diagnosed with fibromyalgia	11 FM patients	Purposive sampling through 2 self-help groups	Focus groups with two interviews	Systematic text condensation	Paper
The lived experience of fibromyalgia in female patients, a phenomenological study	Wuytack and Miller	2011	Belgium	To gain a better understanding of the subjective experience of fibromyalgia, focusing on the personal, occupational and social impact of the condition on patients’ lives	6 FM patients	Recruitment was done through a self-help group	Structured interviews	Thematic framework analysis	Paper
Patients’ experiences of living with and receiving treatment for fibromyalgia syndrome: a qualitative study	Lempp, Hatch, Carville and Choy	2009	UK	To understand patients’ experience of living with this long-term condition, using qualitative interviews.	21 FM patients	Purposive sampling through a rheumatology outpatient clinic	Semi-structured interviews	Content/discourse analysis	Paper
Patients’ and professionals’ views on managing fibromyalgia	Briones-Vozmediano *et al*.	2013	Spain	To examine three aspects of fibromyalgia management: diagnostic approach, therapeutic management and the health professional-patient relationship, to explore areas of the health care process that professionals and patients may consider unsatisfactory	9 health professionals and 12 FM patients	Convenience sample through patient associations and snowballing technique	Semi structured interviews	Discourse analysis	Paper
Understanding the Impact of Fibromyalgia on Men: Findings From a Nationwide Survey	Muraleetharan *et al*.	2018	USA	To understand FM by examining multiple impacts of fibromyalgia on men in regard to interactions in society and the U.S. health system	1163 (805 with FM) patients	Convenience sampling through community health events and online	Qualitative survey	Thematic analysis	Paper
Patients’ quest for recognition and continuity in health care: time for a new research agenda?	Gjengedal *et al*.	2019	Norway	To explore what persons who have been critically ill and persons suffering from long-lasting chronic muscle pain, consider as important research topics, based on their own experiences of illness, and encounters with healthcare services	23 patients (14 with FM)	Recruitment was through two hospitals, physiotherapy outpatient clinics and three patient organisations	Focus groups and phenomenological reflections	Phenomenological reflections	Paper
When pain in the arm is ‘all in the head’: The management of medically unexplained suffering in primary care	Wainwright *et al*.	2006	UK	Not given	14 healthcare workers	Purposive sampling through a previous upper limb disorder study	Interviews	Thematic analysis	Paper
Misunderstandings in prescribing decisions in general practice: Qualitative study	Britten *et al*.	2000	UK	To identify misunderstandings between patients and doctors that have potential or actual adverse consequences for taking medicines	20 GPs and 35 participants (1 FM)	Purposive sampling from GP practices	Semi-structured interviews.	Not given	Paper
Fibromyalgia and the therapeutic relationship: Where uncertainty meets attitude	Hayes *et al*.	2010	Canada	To gain a better understanding of the nature of the patient–provider relationship in fibromyalgia and the underlying knowledge and attitudinal challenges	189 GPs, 139 specialists, two nurses and 18 patients	Purposive sampling through lists, adverts and patient advocate groups	Discussion groups and semi-structured interviews	Grounded theory analysis	Paper
Doctors’ attitudes to fibromyalgia: a phenomenological study	Hellström *et al*.	1998	Sweden	To trace out the meaning structure that guides GPs’ and rheumatologist experiences of and attitudes towards FM and FM patients	10 rheumatologists and 10 GPs	Not given	Interviews	Not given	Paper
Patients’ and practitioners’ views and experiences of chronic widespread pain (fibromyalgia) and its management in the UK and Libya	Abokdeer	2019	England	To capture service users’ and service providers’ views and experiences of fibromyalgia and its management, including their views on the journey to diagnosis, current practice availability, emotional and life experience, information seeking and experiences of seeking and receiving health care	12 FM patients and 53 healthcare professionals	Purposive sampling through a fibromyalgia self-help group and the British Pain Society	Semi structured interviews	Framework analysis	Thesis
Health care communication issues in fibromyalgia: an interpretive description	Thorne *et al*.	2004	British Columbia	To portray how individuals with fibromyalgia (FM) describe interactions with health care providers and to highlight patterns of communication that were perceived as helpful or unhelpful during these encounters	11 FM patients	Purposive sampling through a provincial FM society and newspaper advertisements	Interviews	Constant comparative analysis	Paper
How do patients with fibromyalgia talk about their experience of fibromyalgia?	Agyare	2020	England	To uncover the ways in which patients diagnosed with fibromyalgia talked about their experience of fibromyalgia.	7 FM patients	Snowballing sample from social media and posters in FM clinics	Semi-structured interviews	Foucauldian discourse analysis	Thesis
Meaning, information and online participation along the illness journey: The story for fibromyalgia patients	Chen	2015	USA	To explore the illness journeys of fibromyalgia patients, including how patients’ views of illness and of health evolve over time, what role information plays, and how their participation in online communities, Facebook, Twitter and other social media changes.	23 FM patients	Purposive sample from online discussion forums, twitter, face-to-face support groups, university lists and from a pool of previous survey participants	Semi-structured interviews	Thematic analysis	Thesis
Living With a Contested, Stigmatised Illness: Experiences of Managing Relationships Among Women With Fibromyalgia	Armentor	2017	USA	To explore the various approaches used by individuals with FM to manage their social roles and negotiate their relationships with others including family members, friends, coworkers and medical professionals	20 FM patients	Convenience/snowball sampling using flyers posted in rheumatologists offices, coffee shops and via word of mouth	Semi-structured interviews	Grounded theory	Paper
The complaining women”: health professionals’ perceptions on patients with fibromyalgia in Spain	Briones-Vozmediano *et al*.	2017	Spain	To explore health service providers perceptions regarding fibromyalgia patients in Spain and to analyse possible consequences of these perceptions in terms of how health service providers construct the disease and treat their patients	12 health service providers (1 GP)	Theoretical/ snowball sampling through direct contact with healthcare professionals	Semi-structured interviews	Qualitative content analysis	Paper
How Do Patients with Fibromyalgia Talk about their Experience Of Fibromyalgia?	Agyare	2020	UK	To uncover the ways in which patients diagnosed with fibromyalgia talked about their experience of fibromyalgia.	7 FM patients	Snowball sampling through social media and posters in FM clinics	Semi-structured interviews	Foucauldian discourse analysis	Thesis
Creating meaning in fibromyalgia syndrome	Madden and Sim	2006	UK	To explore the creation of meaning in a medically unexplained disorder, fibromyalgia syndrome (FMS)	17 FM patients	Randomly selected via hospital database	Semi-structured interviews	Induction-abduction method and documentary analysis	Paper
Acquiring a diagnosis of fibromyalgia syndrome: The sociology of diagnosis	Madden and Sim	2016	UK	To explore the experience of diagnosis in FMS and to evaluate the contribution that the negotiated order, viewed alongside other perspectives, can make to an understanding of this experience.	17 FM patients	Purposive sampling from a rheumatology clinic	Semi-structured interviews	Unclear	Paper
MAPS OF MARGINALIZATION: EXPLORING THE HEALTHCARE EXPERIENCES OF MEN AND WOMEN WITH FIBROMYALGIA	Skop	2015	Canada	To explore the retrospective and ongoing healthcare experiences of men and women who have a diagnosis of fibromyalgia (FM).	35 FM patients	Convenience sampling via community support groups	Interviews and focus groups	Grounded theory	Thesis
Patients’ views: improving care for people with fibromyalgia	Egeli *et al*.	2008	Canada	To examine patients’ descriptions of both positive and negative physician encounters, provide patient recommendations for improved care and consider the implications of these findings for nursing practice.	42 FM patients	Sampling method not stated. Patients were recruited through online support groups.	Open-ended questions	Qualitative descriptive approach	Paper
Young pain patients’ experience in primary care. A qualitative study	Hiort, Lindau and Lofgren	2017	Sweden	The purpose was to explore interview data from young adults with long-standing pain about their experience of contacts with caregivers in a primary care setting	11 young adults with chronic pain (4 with FM)	Participants recruited from a young-adult rehabilitation program	Interviews	Thematic content analysis	Paper
The lived experience of fibromyalgia in female patients, a phenomenological study	Wuytack and Miller	2011	Belgium	To gain a better understanding of the subjective experience of fibromyalgia, focusing on the personal, occupational and social impact of the condition on patients’ lives	6 FM patients	Participants recruited through a self-help group	Semi-structured interviews	Thematic framework analysis	Paper
Lived experiences of fibromyalgia	Diviney and Dowling	2015	Ireland	The purpose of this study was to explore the lived experience of fibromyalgia.	9 FM patients	Participants recruited through the social media site ‘FibroIreland’	Open interviews	Lifeworld approach	Paper

## Appendix 3 CASP quality assessment

**Table:**

Study	Year	A1: Was there a clear statement of the aims of the research?	A2: Is a qualitative methodology appropriate?	A3: Was the research design appropriate to address the aims of the research?	A4: Was the recruitment strategy appropriate to the aims of the research?	A5: Was the data collected in a way that addressed the research issue?	A6: Has the relationship between researcher and participants been adequately considered?	B7: Have ethical issues been taken into consideration?	B8: Was the data analysis sufficiently rigorous?	B9: Is there a clear statement of the findings?	C10: How valuable is the research?
Abokdeer	2019	Yes	Yes	Yes	Yes	Yes	Maybe	Yes	Yes	Yes	Valuable
Agyare	2020	Yes	Yes	Yes	Yes	Yes	Yes	Yes	Yes	Yes	Valuable
Armentor	2017	Yes	Yes	Yes	Yes	Yes	Yes	Yes	Yes	Yes	Valuable
Boulton	2019	Yes	Yes	Yes	Yes	Yes	Yes	Yes	Yes	Yes	Valuable
Briones-Vozmediano *et al*.	2013	Yes	Yes	Yes	Yes	Yes	No	Yes	Unsure	Yes	Valuable
Briones-Vozmediano *et al*.	2018	Yes	Yes	Yes	Yes	Yes	No	Yes	Yes	Yes	Valuable
Britten *et al*.	2000	Yes	Yes	Unsure	Yes	Unsure	No	Yes	Unsure	Unsure	Valuable
Chen	2015	Yes	Yes	Yes	Yes	Yes	Maybe	No	Yes	Unsure	Unsure
Chen and Swaminathan	2019	Yes	Yes	Yes	Yes	Yes	No	Yes	Yes	Yes	Valuable
Cooper and Gilbert	2017	Yes	Yes	Yes	Unsure	Maybe	No	Yes	No	Maybe	Not valuable
Diver	2012	Yes	Yes	Yes	Yes	Yes	Yes	Yes	Yes	Yes	Valuable
Diviney and Dowling	2015	Yes	Yes	Yes	Unsure	Maybe	No	Yes	Unsure	Unsure	Unsure
Durif-Bruckert, Roux and Rousset	2014	Yes	Yes	Yes	Yes	Yes	No	Yes	Unsure	Unsure	Unsure
Egeli *et al*.	2008	Yes	Yes	Yes	Yes	Unsure	No	Yes	Unsure	Yes	Valuable
Gjengedal *et al*.	2019	Yes	Yes	Yes	Yes	Yes	No	Yes	Yes	Yes	Valuable
Hayes *et al*.	2010	Yes	Yes	Yes	Yes	Yes	No	Yes	Yes	Yes	Unsure
Hellström *et al*.	1998	Yes	Yes	Yes	Yes	Unsure	Unsure	No	No	No	Not valuable
Hiort *et al*.	2016	Yes	Yes	Yes	Yes	Yes	No	Yes	Yes	Yes	Valuable
Lempp *et al*.	2009	Yes	Yes	Yes	Yes	Yes	Yes	Yes	Yes	Yes	Valuable
Madden and Sim*	2016	Yes	Yes	Yes	Yes	Yes	Yes	Yes	Yes	Yes	Yes
Madden and Sim*	2006	Yes	Yes	Yes	Yes	Yes	Yes	Yes	Yes	Yes	Valuable
Muraleetharan *et al*.	2018	Yes	Yes	Yes	Yes	Unsure	No	No	Yes	Yes	Valuable
Paulson, Norberg and Danielson	2002	Yes	Yes	Yes	Yes	Yes	No	Yes	Yes	Yes	Valuable
Reibel and Pearson	2017	Yes	Yes	Yes	Unsure	Yes	No	No	Yes	Yes	Unsure
Rodham, Rance and Blake	2010	Yes	Yes	Yes	Yes	Yes	No	Yes	Yes	Yes	Valuable
Skop	2015	No	Yes	Yes	Yes	Yes	Yes	Yes	Yes	Yes	Valuable
Thorne *et al*.	2004	Yes	Yes	Yes	Yes	Yes	No	Yes	Yes	Yes	Valuable
Undeland and Malterud	2009	Yes	Yes	Unsure	Yes	Yes	No	No	Yes	Yes	Valuable
Wainwright *et al*.	2006	No	Yes	Yes	Yes	Yes	No	Yes	Yes	Yes	Unsure
Wuytack and Miller	2011	Yes	Yes	Yes	Yes	Yes	Unsure	Yes	Yes	Yes	Valuable

## Appendix 4 Author career backgrounds

**Table:**

Author	Career background
**Ms Ailish Byrne**	MPsych; MSc Applied Health Research. Trial Coordinator, York Trials Unit. Research interests include: chronic pain conditions, women’s health, body schema and interoception.
**Dr Katherine Jones**	BSc Sports Science with coaching; MSc Clinical Exercise Physiology; PhD Clinical Exercise Physiology. Research Fellow, University of Warwick. Research interests include: the role of exercise prescription in the management, treatment and prevention of chronic diseases, with a particular interest in inflammatory bowel disease (Crohn’s disease and ulcerative colitis)
**Dr Michael Backhouse**	BSc(Hons) Podiatry; PhD Medicine; Associate Professor, University of Warwick. Fellow of Royal College of Podiatry; Fellow of Faculty of Podiatric Medicine, Royal College of Physicians and Surgeons (Glasgow). Research interests include: effectiveness of interventions for foot and ankle pathology in people with long-term conditions with a particular interest in rheumatic and musculoskeletal disease.
**Ms Fiona Rose**	BA Counselling Studies; MSc Public Health; Trial Coordinator, York Trials Unit. Previous occupation within frontline mental health services. Research interests include: mindfulness, mental health and well-being and qualitative evidence synthesis.
**Ms Emma Moatt**	BA Applied Social Sciences; MSc Health Promotion. Trial Coordinator, York Trials Unit. Research interests include: orthopedic and plastic surgery along with emergency medicine research.
**Professor Christina van der Feltz-Cornelis**	MD Medicine; MedSc Psychiatry; Member of the Dutch Society of Psychiatrists and registered as psychotherapist; Reg VvE Epidemiology; PhD Medicine; SMWBO Epidemiology; International Fellow of the APA; Full license to practice gmc/ Honorary Consultant Psychiatry. Chair of Psychiatry and Epidemiology, University of York. Research interests include: better understanding, diagnosis and treatment of common mental disorders and the interface of somatic symptoms.
