# Supplementary material for: Patient and primary care practitioners’ perspectives on consultations for fibromyalgia: a qualitative evidence synthesis
Source: Prim Health Care Res Dev. 2023 Sep 26;24:e58. doi: 10.1017/S1463423623000506 (PMC10540196; doi:10.1017/S1463423623000506)
Supplement: Supplementary file 1 [file phcsup.zip › S1463423623000506sup001.pdf]

Code	GP or patient	Quote	Author	Page
Lack of continuity of care	Patient	"They had to repeat their symptoms over and over again, and consequently felt here was no continuity of health care."<br><br>"There was a new doctor that I had never met before – I usually see Doctor X but he was off sick, and then the next time I was there he was no longer employed there – so I had to talk to yet another doctor and it's the same story again".<br><br>"Participants also mentioned their frustration regarding the number of specialists they had to consult, as they were frequently passed from GPs to other healthcare professionals, which made finding a solution within primary and secondary care systems more difficult."<br><br>"Basically first I went to my GP and I kept going kept going and I saw a different GP, she did a lot of different blood tests."	Paulson, Norberg & Danielson, 2002<br><br>Paulson, Norbery & Danielson, 2002<br><br>Abokdeer, 2019<br><br>Agyare, 2020	p.90<br><br>p.90<br><br>p.92<br><br>p.99
Exhaustive process	Patient	"Her GP discussed the possibility of FM over two decades before she was officially diagnosed"<br><br>"I kept going back to the GP over and over again with the pain and he sent me for various tests but couldn't find anything wrong".	Boulton, 2019<br><br>Boulton, 2019	p.813<br><br>p.812
Lack of time	Patient	"In primary care the main concern was perceived lack of time by GPs in their consultations"<br><br>"My GP is understanding. He does not have much time that is why I asked to see a rheumatologist. He is so fast, only 5 minutes 'and what do you have? More prescription here you are, bye bye'".<br><br>"When seeking help in primary care for their symptoms, the men explained that because of the physicians lack of scheduled time, it was difficult to find a receptive listener".	Lempp et al., 2009<br><br>Lempp et al., 2009<br><br>Paulson, Norberg & Danielson, 2002	p.7<br><br>p.7<br><br>p.90
Biomedical model over subjective	Patient	She had been to her GP and was not satisfied with the biomedical reason for her symptoms that had been given".	Diver, 2012	p.197

experience of symptoms		"She consulted her GP who initialled diagnosed her with arthritis but when her symptoms did not resolve she felt this was an inadequate explanation".<br><br>"'I went to my GP who sent me for x-rays and blood tests and that. And they found some arthritis in my spine. Nothing much. It wasn't a lot but they just put it down to 'it it's the arthritis''. She now thought that whilst this would have explained her pain symptoms it did not explain the tiredness"<br><br>"Diagnosis offered by the GP often incorporated informants initial explanations of the symptoms, which included everyday life events. However, the GP also explained symptoms in terms of concurrent illnesses, such as osteoarthritis, asthma, and diabetes".<br><br>"Informants had often used such explanations within their own search for meaning in their symptoms, these explanations had normally been rejected as inadequate to explain the degree of biological disruption".<br><br>"Informants frequently reconsidered these explanations where proffered by the GP".<br><br>"I tried for two years, kept going to my doctors saying I got this pain in my legs and my arms and he [GP] kept, oh it'll go off, it's your age, you know you're going through the crisis. And I believed all that."	Diver, 2012<br><br>Diver, 2012<br><br>Madden & Sim, 2016<br><br>Madden & Sim, 2016<br><br>Madden & Sim, 2016<br><br>Madden & Sim, 2016	p.198<br><br>p.194<br><br>p.97<br><br>p.97<br><br>p.97<br><br>p.97
	GP	"It's very subjective, so this is just based on a faith in patients saying […] 'It hurts, my [..] is sore". But you don't have a measure of ow much you can press on the [tender] point."	Hayes et al. 2010	p.387
Lack of knowledge	Patient	"Not all GPs and specialists applied the same criteria to treat the chronic widespread pain of FMS. Because of the participants dissatisfaction with the aforementioned issues, their relationship between healthcare professionals was sometimes strained."<br><br>"It was also felt that GPs and healthcare professionals needed to update their knowledge to develop a proper understanding of their condition."<br><br>"If General Practitioners understood it yes, it would be a lot better for everybody and it would help I think more and more people with fibromyalgia to – not get better but of course feel better at least you know."<br><br>"Participants also discussed their experiences of being misdiagnosed, and their concerns about the development of expertise understanding and management of FMS in the UK. This appeared when	Abokdeer, 2019<br><br>Abokdeer, 2019<br><br>Diviney & Dowling, 2015<br><br>Abokdeer, 2019	p.92<br><br>p.92<br><br>p.5<br><br>p.76

		patients debated their GPs and consultants, and found it difficult to be involved in the management of their condition, because of the lack of understanding of the cause of their pain.”		
		“If there were more GPs, I think, who were more, like, more knowledgeable of the condition, it would help.”	Abokdeer, 2019	p.77
		“Eight out of twelve participants were dissatisfied with how they were treated by GPs, professionals, and therapists, due to the lack of understanding of their condition.”	Abokdeer, 2019	p.78
		“The GP didn’t have any idea, the GP didn’t know about everything.”	Abokdeer, 2019	p.78
		“The GP, you know, didn’t have a clue about fibromyalgia, didn’t have a clue about what my pain was.”	Abokdeer, 2019	p.81
		“The overall theme emerging from discussions with the participants was that understanding the condition and gaining knowledge about it, are exclusive responsibilities for the GPs and specialists.”	Abokdeer, 2019	p.82
		“It’s very stressful because when you go to the GP I think you want them to know. You expect them to be able to pinpoint at what you’re suffering from. So clearly if they, if they don’t know. I think it’s almost like they weren’t, I wanna say the fact they won’t take me seriously but I think they don’t know.”	Agyare, 2020	p.114
	GP	“It does have some criteria, and its complex, and we definitely don’t understand it as well as we need to.”	Hayes et al. 2010	p.387
		“Even among those who believed in Fibromyalgia, there was uncertainty about whether it was essentially a biomechanical or psychosocial phenomenon.”	Wainwright et al. 2006	p.83
		“It’s very subjective, so this is just based on a faith in patients saying […] ‘It hurts, my [..] is sore”. But you don’t have a measure of ow much you can press on the [tender] point.”	Hayes et al. 2010	p.387
		“I usually find that it is something you think of when you are slightly scratching your head. Someone who has got more muscle rather than joint problems, although they often seem to initially present with a joint that is causing the problem and then you start talking to them and ‘actually I have got all these other things wrong with me”.	Wainwright et al. 2006	p.83
Patients physical appearance not matching their	Patient	“GPs and healthcare professionals observed patients physically, which was not mirrored by how the patients felt inside.”	Abokdeer, 2019	p.80

internal experience		"Participants described how they were questioned at times about whether they genuinely had been ill and suffering with pain by people at work, and even by GPs, as the result of looking physically well."	Abokdeer, 2019	p.89
Lack of effective treatments	Patient	I found myself unable to stand Laroxyl or antalgics anymore so I went to see my regular practitioner who said to me 'what do you want me to do'."	Durif-Bruckert, Roux & Rousset, 2014	p.2588
		"They all prescribe different medication because they don't really know what it is. The medication they give you is harmful to begin with, they make you worse than you are".	Hayes et al, 2010	p.388
		"I went back to him [primary physician] and I said "We've got to find something". He said "Well maybe we could put you on Xanex" and I said "That's not solving the problem".	Reibel & Pearson, 2017	p.71
		"I think as time passes that it's more to do with the stress and depression. And the GP she suggested I try anti-depressants, but I'd rather try counselling than that. I've had aches and pains before but this is different".	Diver, 2012	p.217
		"In her talk she also suggests that during her discussions with her GP about how to manage Fibromyalgia once diagnosed, anxiety and pain were not adequately addressed. It could be argued that participants may feel as though their emotional as well as physical needs are not fully spoken about during their treatment talks with GPs."	Agyare, 2020	p.119
		"I tried a collage of different medications, my new doctor was very frustrated with me and I was equally frustrated with him."	Skop, 2015	p.206
	GP	"You're getting yourself into a vortex of side effects and perhaps drug overuse and misuse and abuse."	Hayes et al., 2010	p.388
		"There's a few drugs that have been tried and nothing seems to work too well and that's it."	Hayes et al., 2010	p.387
Lack of confidence in GP	Patient	"They were influenced by their inability to live their 'normal' lives and began to lose confidence in their own explanations for their symptoms as well as those provided by their GP."	Diver, 2012	p.196
		"Failure by their GP to diagnose this complaint led the majority to doubt the ability for their GP to manage their symptoms".	Diver, 2012	p.207
		"GPs and healthcare professionals were described as not confident in the management of FMS".	Abokdeer, 2019	p.81

Perceived importance of diagnosis	Patient	"Her GP discussed the possibility of FM over two decades before she was officially diagnosed."	Boulton, 2019	p.813
		"'She said that we would need to run a whole bunch of tests to make sure it's not something else first. As I got older it's got to the point where I wanted an official diagnosis'".	Boulton, 2019	p.813
		"I had a primary doctor and I kept telling him I didn't feel right. I just didn't feel right at all. I hurt, my skin was crawling and I just didn't feel right. So, he [primary physician] goes "Well, we could check into it, you know uh, you might have chronic pain or it might just be fibromyalgia"	Reibel & Pearson, 2017	p.70
		"He said I may be having symptoms of that. So I said 'Is there anything you can do to find out? [primary physician reply] 'No, there is no test for it', and I said 'Ok'." The appointment ended abruptly as she head her physician say "No there is no test for it".	Reibel & Pearson, 2017	p.70
		"One woman quoted her GP's attitude to her request for a diagnosis: 'It can wait until you claim disability benefits'. She had no intention of leaving work. When Fibromyalgia was confirmed by a rheumatologist years later, she had burst into tears: 'Finally a diagnosis! Finally I know what is wrong with me!"	Gjengedal et al. 2019	p.981
		"This period of chaos appeared to be shorter for those participants [..] for whom the diagnosis of FMS was suggested early on by their GPs".	Diver, 2012	p.223
		"I said to the general physician, 'This chiropractor thinks I have Fibromyalgia' and he said 'I do too, I just did not want to make that diagnosis because I was afraid it would impact your health insurance in the future'. So that was really shocking to me, and I kind of understood later what he was talking about, but I still didn't think that was right at all not to tell me what he thought my problem was"	Chen & Swaminathan, 2020	p.141

	GP	“Well if she seems to have use for a diagnosis to achieve her goal, I’ll gladly help her”<br><br>“One GP started his narrative by saying: “In meeting an FM patient I do not aim at making a diagnosis. I think this intrudes on my option to develop a true dialogue”.<br><br>The GPs seemed comparatively less involved in diagnostic precision, perhaps because they had the option to refer the patient to a rheumatologist.”<br><br>“Discrepancies found in the tendency between GPs and Rheumatologists to make a diagnosis can be due to the options that the diagnosis gave access to. The rheumatologists seemed to be comparatively more anxious to make a diagnosis. […] the interviews gave the impression that the GPs were more accustomed to living with uncertainty and with a less structured practice than hospital clinicians”.<br><br>“Others were happy to use it [FM diagnosis], not least because it provided a bridge between bio-mechanical and psychosocial explanations and avoided invalidating the patient”.<br><br>“It is a bridge between the two isn’t it [biomechanical and psychosocial]? Saying ‘yes you have got physical symptoms and they are real.”<br><br>“As well as reassuring the patient, the ascription of a diagnosis of Fibromyalgia may also mark a transition in the consultation, effectively bringing the ‘work up’ to a close and leading to the deployment of treatment options.”<br><br>“The GPs attitudes to the diagnostic procedure were comparatively less compelling.”<br><br>“…once I give that label it means I am ready to say to the patient, lets stop doing tests. Let’s stop sending you lots of different doctors looking for a cure, lets accept if we can that this is whats going on and now lets try and make you better.”<br><br>“If I give that label to you that says: I believe that you are in pain. This isn’t made up and a ploy to get sick notes. ‘This is real pain and I believe you and I am going to give you a diagnosis.’ And this is a bridge between the mental aspects of it and the physical aspects.’	Hellstrom et al. 1998<br><br>Hellstrom et al. 1998<br><br>Hellstrom et al. 1998<br><br>Hellstrom et al. 1998<br><br>Wainwright et al. 2006<br><br>Wainwright et al. 2006<br><br>Wainwright et al. 2006<br><br>Hellstrom et al., 1998<br><br>Wainwright et al. 2006<br><br>Wainwright et al. 2006	p.234<br><br>p.235<br><br>p.234<br><br>p.235<br><br>p.82<br><br>p.82<br><br>p.82<br><br>p.234<br><br>p.82<br><br>p.82
Societal expectation of the GP	Patient	“Although the medical explanation contradicted their personal beliefs, informants were willing to take the diagnosis seriously, because of their trust in, and high expectations of, their GP.”	Madden & Sim, 2016	p.97

		"You go blindly on, you're not really satisfied, but then I thought well there's people a lot worse off than me. If that's what she [GP] says it is, that's what it must be."	Madden & Sim, 2016	p.97
GP's attitudes towards patients (doubt, scepticism, unkind)	Patient	"The men perceived that they were not being respected and not quite taken seriously, which upset them."<br><br>"They felt neglected when treated by health care staff in foremost primary care and general hospital, which they felt were not committed and who were in fact sometimes unkind".<br><br>"Even if the staff believed their pain was genuine, feelings of being looked upon as exaggerating their pain experience was expressed".<br><br>"I found myself unable to stand Laroxyl or antalgics anymore so I went to see my regular practitioner who said to me 'what do you want me to do'."<br><br>"This path is characterised by a strong feeling of disappointment, rejection, and incompetence (very often involving shame and humiliation) regardless of the status of the doctor the patient has consulted, general practitioners or well-known specialists"<br><br>"In addition some voiced concern that their GP might continue to doubt their credibility"<br><br>"[the GP] failed to appreciate the impact that FMS had on them".<br><br>"They still didn't want to believe it…I had to change GP. I err went through a very bad time with the GP, who just said I didn't have anything wrong with me, still wouldn't, you know, didn't want to accept the diagnosis"<br><br>"She never consulted her GP for other symptoms, fearing dismissal"<br><br>"I assumed, he [GP] kept thinking well I'm going down here with pains, nothing has come back on the blood results, nothing has come with the x-rays, so there's nothing wrong with me."<br><br>"When visiting the primary care health centre in the early stages, the men expressed feeling of being treated politely. As time went on and no cure was found, it was as If the health staff lost	Paulson, Norbery & Danielson, 2002<br><br>Paulson, Norbery & Danielson, 2002<br><br>Paulson, Norbery & Danielson, 2002<br><br>Durif-Bruckert, Roux & Rousset, 2014<br><br>Durif-Bruckert, Roux & Rousset, 2014<br><br>Diver, 2012<br><br>Diver, 2012<br><br>Rodham et al. 2010<br><br>Undeland & Malterud, 2007<br><br>Madden & Sim, 2016<br><br>Paulson, Norbery & Danielson, 2002	p.93<br><br>p.93<br><br>p.91<br><br>p.2588<br><br>p.2588<br><br>p.207<br><br>p.244<br><br>p.71<br><br>p.252<br><br>p.99<br><br>p.91

		interest in them, 'the quicker one leaves the better'. All men felt enormously alone as the staff seemed to be neither interested nor involved in them."		
		"Time and time again the men received prescriptions for medication without having a proper conversation with the physician".	Paulson, Norberg & Danielson, 2002	p.91
		"They considered that were 'fobbed off' with medication."	Diver, 2012	p.244
		"My brother in law is a GP, he was the one who told me I had Fibromyalgia most likely, but he also said he did not want to have me as his patient anymore because he wouldn't be able to help me anyway….that was a bit of a slap in the face!"	Wuytack & Miller, 2011	p.5
		"The GPs just don't wish to know."	Abokdeer, 2019	p.81
		"That particular GP, you know they have no time. Sympathy not there, empathy not there at all."	Agyare, 2020	p.100
		"I think the impression I get from GPs they just want people to get on with their life and sort themselves out."	Agyare, 2020	p.110
		"for example, Angelika booked frequent appointments with her family physician in order to assess and treat new symptoms; she did not know if these symptoms were part of FM or indicative or an undiagnosed co-morbid condition. 'I kept going to the doctor every two days and he said 'if you keep coming here I'm going to put you in a crazy house'. …Like to be institutionalized because he didn't want to hear any more of my nonsense. Because he took me as crazy."	Skop, 2015	p.158
		"Participant 1 was told by her GP: 'You have to educate yourself and you have to just heal yourself."	Agyare, 2020	p.5
	GP	" I think that the bottom line is no one really wants to look at it. It's a lot of work, it's very unrewarding, you don't really see any concrete results. It's always like a constant decline, so it becomes very unsatisfying as a result".	Hayes et al. 2010	p.389
		"The interviewees, particularly the GPs, began their descriptions with a sigh."	Hellstrom et al., 1998	p.235

		"I'm reluctant as far as this sort of thing is concerned, but I have to admit that it comes down to my own prejudice. I hold it against this sort of patient to a certain degree, they're soft, you have to put pressure on them so that they will liven up their act/…/I think that in cases of women with fibromyalgia you're conditioned to think twice before granting them work leave."<br><br>"They are patients that…towards them whom I feel rejection, I have to admit."	Briones-Vozmediano, 2018<br><br>Briones-Vozmediano, 2018	p.1682<br><br>p.1682
Typecasting/judgements of FM (condition & patients)	GP	"Identifying patients who would be seen as 'worth it' was identified as a need by participants."<br><br>"Is there a screening method, a psychological profiling of sorts that say: this is a patient worth working with and these are patients that [aren't]."<br><br>"The other characteristic of this group of patients, from my experience is, that they do tend to do the rounds of doctors looking for a cure, and they do tend to have more tests than is good for them."<br><br>"Doctor:' I think he loves taking medicines actually. For a young man, 10 years younger than me, he's had more medicines than I've had hot dinners. So I can only suppose that he – he likes having them. Something which proves he's ill."<br><br>"They have a lot of complaints about their personal lives – either with their partner or problems at work. We see a lot of cases of women with current or past relationship issues. We also get those who have had bad experiences in their lives…we've even had cases of domestic violence."<br><br>"They feel mentally like no one understands them, and this is part I think, of the characteristics of the profile of fibromyalgia…that wherever they go no one pays attention."<br><br>"It's really not so bad, nor would it justify sick leave. It's not the same when a woman in that condition as to be let off work as it is for a man who works on a building site with a lumber problem is it? You let him off and that's that."	Hayes et al. 2010<br><br>Hayes et al. 2010<br><br>Wainwright et al. 2006<br><br>Britten et al. 2000<br><br>Briones-Vozmediano et al, 2018<br><br>Briones-Vozmediano et al, 2018<br><br>Briones-Vozmediano et al., 2018	p.389<br><br>p.389<br><br>p.83<br><br>p.486<br><br>p.1681<br><br>p.1682<br><br>p.1682

Chaos	Patient	"Once diagnosed chaos narratives were dominated by stories of how when they went to see their GP they didn't appear interested in listening to their plight".	Diver, 2012	p.244
FM is purely psychological	Patient	"For five years I sought and was told, "It's in your mind. It's your imagination. Started with general practitioners, all the way up to psychologists."<br><br>"They described their experiences of other people's perceptions of Fibromyalgia being a non-medical condition, especially when talking about their experiences with their GPs."<br><br>"Many participants GPs or other health professionals constructed Fibromyalgia as a made-up condition that existed in their patients's minds and implied that it was not caused by any physical condition".<br><br>"Participants constructed how health professionals (mainly GPs) constructed Fibromyalgia within a psychological discourse."	Armentor, 2017<br><br>Agyare, 2020<br><br>Agyare, 2020<br><br>Agyare, 2020	p.467<br><br>p.100<br><br>p.100<br><br>p.103
	GP	"For the sceptics, fibromyalgia is at best a misleading term which puts a physiological gloss on psychological problems, and at worst a complete fiction which medicalises 'normal' aspects of physical existence."	Wainwright et al. 2006	p.83
FM isn't real	Patient	"If you had a GP that actually believed in it, it would be absolutely brilliant."<br><br>"Some GPs I know don't believe it, believe in it."<br><br>"Finding a doctor who believed in it, which was quite hard – a GP."<br><br>"The participants talked about experiencing resistance towards their doctors and GPs and talked about trying to convince them that they were ill."	Abokdeer, 2019<br><br>Abokdeer, 2019<br><br>Abokdeer, 2019<br><br>Agyare, 2020	p.86<br><br>p.97<br><br>p.80<br><br>p.102
	GP	"Attitudinal issues began with physicians questioning the validity of Fibromyalgia itself"<br><br>"I'm not convinced, how can I convince somebody? That's my problem with Fibromyalgia, I'm not convinced at all."	Hayes et al., 2010<br><br>Hayes et al., 2010	p.387<br><br>p.387

		"Many informants were sceptical about the status of fibromyalgia as a legitimate disease category."<br><br>"I think giving it a label that actually has no justification I think is misleading to the patient and I actually feel quite strongly about that."<br><br>"For the sceptics, fibromyalgia is at best a misleading term which puts a physiological gloss on psychological problems, and at worst a complete fiction which medicalises 'normal' aspects of physical existence."<br><br>"Some participant GPs believed that it is not a diagnosable condition, in contrast with those who expressed knowledge and skill in diagnosing the disorder".	Wainwright et al. 2006<br><br>Wainwright et al. 2006<br><br>Wainwright et al. 2006<br><br>Hayes et al., 2010	p.82<br><br>p.83<br><br>p.83<br><br>p.389
Lack of communication	Patient	"While men with FM still rely on seeing their primary care provider shortly after experiencing symptoms, there is a communication gap between men with FM and their HCPs".<br><br>"Time and time again the men received prescriptions for medication without having a proper conversation with the physician."<br><br>"Entrusting her physicians word and medical expertise, Kay left the appointment with an 'ok' and without any clear answers for what was causing her pain or how to manage the pain."<br><br>"Yes but it was a bit unclear cos' he just said 'yes, you'll stay on here since you've probably got fibromyalgia". I just went 'so I've got fibromyalgia!' and he said 'we mustn't jump to conclusions' so I say to him 'why must I come here, have I got it or not' kind of thing. He was so….he never gave straight answers to questions."<br><br>"When seeking help in primary care for their symptoms, the men explained that because of the physicians lack of scheduled time, it was difficult to find an receptive listener"<br><br>"Once diagnosed chaos narratives were dominated by stories of how when they went to see their GP they didn't appear interested in listening to their plight".<br><br>Patient: '[..] I wanna come off the painkillers […] I'll probably come off them for about four, three or four, days so I can get my pains in my joints, so when I go to the s- see the er arthritis specialist then he could s- suss out what my joints are like'.<br>Interviewer: 'Right, yeah. Mmm	Muraleetharan et al. 2018<br><br>Paulson, Norberg & Danielson, 2002<br><br>Reibel & Pearson, 2017<br><br>Hiort, Lindau & Lofgren (2017)<br><br>Paulson, Norberg & Danielson, 2002<br><br>Diver, 2012<br><br>Britten et al., 2000	p.956<br><br>p.91<br><br>p.70<br><br>p.91<br><br>p.90<br><br>p.244<br><br>p.486

		Patient: 'It's if –err without the painkillers – if I've got too many painkillers he can't asses what's in my – what my pains are like'<br>Interviewer: Mmm. Did the Doctor tell you to do that or were you just doing it yourself?<br>Patient: 'No, I'll do it – I'm gonna do it myself'."		
		"Summary of misunderstanding. Mr C has an appointment to see a rheumatologist in a few weeks time. He intends to stop taking all his drugs a few days before seeing the consultant, but he does not tell Dr D [GP]. Mr C is worried about taking too many painkillers. Dr D is unaware of this and thinks that he likes taking medication."	Britten et al., 2000	p.486
		"Well, I've got a hiatus hernia apparently that I didn't know that I'd got. [GP] wants to get my stomach right before he carries on with the fibromyalgia. But my stomach isn't the problem, it's the fibromyalgia that's the problem. But he won't refer me back yet, he says I've got to wait. But that's [hiatus hernia] not bothering me. I can cope with that, you can go on a special diet, you can eat what you can, you've got to be careful but I can cope with that. It's the rest of it I can't cope with and that's what I can't get through to him."	Madden & Sim, 2006	p.2969
	GP	"Physicians often described the therapeutic relationship as unidirectional, with patients expected to follow physicians instructions"	Hayes et al. 2010	p.389
Positive GP behaviours (listening, validating, supportive, understanding, pro-active)		"One participant described a particularly sensitive family physician as offering any information he could, sometimes about aspects she had not yet considered. For example, he offered to send her mother some printed information on FM, encouraging the mother to discuss the disease with her own doctor so that she could become more sympathetic to the daughters condition."	Thorne et al. 2004	p.35
		"I discussed it with my GP, we'd discuss it regularly and we'd see where my illness was at. I'd tell her I'd read something and she paid good attention to me."	Durif-Bruckert, Roux & Rousset, 2014	p.2590
		"and it felt…[as if] he took me seriously, [and he] regarded me as a person capable of making my own thoughts and reaching my own conclusions, and I think that's what made it safe for me to take the diagnosis to him. I don't think I would have done that with a lot of other doctors"	Cooper & Gilbert, 2017	p.347
		"I think what made it safe for me to take the diagnosis to the doctor was that I'd build up quite a solid relationship with him….And I just felt that he was very engaging".	Cooper & Gilbert, 2017	p.347
		"The majority (7/12) provided positive comments, e.g: that the GP was sympathetic or supportive"	Lempp et al. 2009	p.7
		"My GP has always been supportive."	Abokdeer, 2019	p.77

		"Hope's female [family] physician expressed sympathy by stating 'I understand how you feel'. This expression of empathy provided Hope with a sense of validation and legitimacy."<br><br>"The only doctors that seem supportive of me is my family doc and specialist".<br><br>"Anyways my doctor has been wonderful, I still present with different symptoms and he still will test me for those symptoms to see what might be causing them. At one point I said to him 'I'm coming back to you all the time with these symptoms and we're finding nothing'. I said 'I really feel like I'm wasting your time' and he said 'that's my job'. He said 'it's not your job to assume it's your fibromyalgia, it's my job to figure out whether it is your fibromyalgia'…he said 'you have fibromyalgia but you can have anything else too.' He said 'that's my job to figure out what it is, and if it is fibromyalgia, fine, but I'm going to make sure'. So I have an awful lot of confidence in my doctor, that's my family doctor.'<br><br>She stated 'I've had good experiences, I've never had anybody say 'it's all up there in your rectum'. Crazy things like that. No I've never had. I have a good family doctor."	Skop, 2015<br><br>Muraleetharan et al., 2018<br><br>Skop, 2015<br><br>Skop, 2015	p.180<br><br>p.956<br><br>p.248<br><br>p.257
GPs as 'gatekeepers'/an information source	Patient	"They feared they might be denied access to future treatments and the possibility of a cure. In this way they were demonstrating how they perceived their GPs to be 'gatekeepers'."	Diver, 2012	p.207
Patients as self-advocates	Patient	"One woman brought information about FM to her general practitioner to assist with reaching a diagnosis."<br><br>"Michelle also showed a clear sense of wanting more information, both at the point of consulting with her endocrinologist and her General Practitioner"<br><br>"I just says to him [GP] I want to see a Rheumatologist because I want to know what is wrong. Couldn't see how I could get better if I didn't know what was going on".<br><br>"As soon as the potential diagnosis of FM has been suggested by her GP she had searched for literature at the library that might help her understand and manage her symptoms".<br><br>"I just couldn't cope with this feeling tired all the time and I know that there was something else wrong so I thought no, I'll go down and see my GP and said 'look there's something else going on".	Egeli et al. 2008<br><br>Cooper & Gilbert, 2017<br><br>Diver, 2012<br><br>Diver, 2012<br><br>Madden & Sim, 2016	p.365<br><br>p.345<br><br>p.200<br><br>p.208<br><br>p.95

		"The participant talked about actively seeking out a medical explanation for her symptom experience by constructing herself as being persistent by visiting her GP practice multiple times and seeing more than one GP within her diagnostic process until one GP conducted the blood tests."	Agyare, 2020	p.99
		"The participants talked about experiencing resistance towards their doctors and GPs and talked about trying to convince them that they were ill, hence the multiple visits, long diagnostic journeys and insistence on referrals to other medical specialists."	Agyare, 2020	p.102
		"I went to the GP and I said 'I want to go and see a rheumatologist."	Agyare, 2020	p.105
		"It was his mother who found information about fibromyalgia online; P13 thought it sounded plausible and brought it up to his general practitioner, which subsequently led to the diagnosis."	Chen, 2015	p.195
		"The participants described that they often asked to be referred to a consultant, as the primary care was unable to offer them something that could help to maintain daily living activates."	Abokdeer, 2019	p.76
		"Participant 1 goes on to challenge her GPs perspective: 'no you have to understand it, this is a proper medical problem and although you can't help me with medication there are other ways you can help me'."	Agyare, 2020	p.104
Collaborative relationship	Patient	"We didn't like each other, but he was stuck with me and I was stuck with him so I had to decide how I was going to figure out the relationship with him…and I bought him a book on fibromyalgia and I put it on his desk and said 'you read it and I'll come back and we'll talk about it.' He wasn't too impressed with me at the time, but now we have a much better relationship and he's listening to me, and it's working…he accepts me now and we sit down and do things together."	Skop, 2015	p.206
		"My understanding is that today there are some doctors who refuse to acknowledge this syndrome exists and some that have no training in dealing with it. It was very helpful to me that my [family] doctor asked me to tell her that if I ever felt she was treating me like someone who had too many symptoms and therefore without merit, not to be afraid to bring it to her attention as this can happen often with patients with fibromyalgia."	Skop, 2015	p.249
		"I've put him up on a pedestal [family doctor] because he's not only my family doctor – he's my councellor, a friend….he belongs on a pedestal as far as I'm concerned."	Skop, 2015	p.256
		"my GP, some of the GPs at my surgery don't really understand it very well so I think it's been like a learning curve for the both of us.'"	Agyare, 2020	p.107

	GP	"…once I give that label it means I am ready to say to the patient, lets stop doing tests. Let's stop sending you lots of different doctors looking for a cure, lets accept if we can that this is whats going on and now lets try and make you better."	Wainwright et al. 2006	p.82
Impact of interactions	Patient	"They pointed out that the lack of support and understanding of people with FM with GPs and HCPs led to anxiety and depression."<br><br>"She expressed that finding a GP who accepted her condition proved to be a crucial element in shaping her illness experience and outcomes positively."<br><br>"Peanuts partly attributed her positive experiences to her doctors legitimation of FM."	Abokdeer, 2019<br><br>Cooper & Gilbert, 2017<br><br>Skop, 2015	p.100<br><br>p.347<br><br>p.257
Societal expectations of patients	Patient	"They considered themselves to be fulfilling social and cultural obligations by attending their GP and being compliant with tests and treatments offered and yet their situation remained the same or worsened."<br><br>"For all the participants the next step in 'normal illness management' was to seek medical opinion, and this was from a GP."<br><br>"Respondent's were most likely to visit a family physician/primary care physician [] to discuss FM, but were more likely to wait 1-6 months before going [] rather than going right away. The respondents flagged the reason for not going to a HCP right away was due to the potential of misdiagnosis or dismissal of male FM patients, citing a general societal belief that a mean needs to seem strong and 'tough it out'."	Diver, 2012<br><br>Diver, 2012<br><br>Muraleetharan et al., 2018	P,224<br><br>p.192<br><br>p.955
Lack of practical information from GP	Patient	"There's not much information as to what's available. Even support groups, you've got to go and find them yourself on the internet. There's no publicity in the GPs surgery or anything. Just like some kind of advice place that would tell you what's available, even if it is the NHS or medication, but give you alternative therapies, you know."	Abokdeer, 2019	p.97
'GP shopping'	Patient	"If I don't like that GP I'll keep going. I'll find somebody else."<br><br>"The participant talked about actively seeking out a medical explanation for her symptom experience by constructing herself as being persistent by visiting her GP practice multiple times and seeing more than one GP within her diagnostic process until one GP conducted the blood tests."	Chen, 2015<br><br>Agyare, 2020	p.225<br><br>p.99
	GP	"The other characteristic of this group of patients, from my experience is, that they do tend to do the rounds of doctors looking for a cure, and they do tend to have more tests than is good for them."	Wainwright et al., 2006	p.83
Patients understanding of FM	Patient	"She was disappointed, because she knew the word 'fibromyalgia' would cling to her, even if she died of cancer or a heart attack."	Undeland & Maltrund, 2007	p.252

GPs unable to help	Patient	"Their GP implied that they could do little to help them."	Agyare, 2020	p.120
	GP	"If I can't' help her, I refer her to a rheumatologist".<br><br>"No matter what you give them, the pain doesn't go away."<br><br>"They are in pain, you give them something for the pain and: "it doesn't do me any good…it relieved the pain a little but the pain has come back", "you gave me this and the pain comes back".<br><br>"You're getting yourself into a vortex of side effects and perhaps drug overuse and misuse and abuse."<br><br>"There's a few drugs that have been tried and nothing seems to work too well and that's it."	Hellstrom et al. 1998<br><br>Briones-Vozmediano et al. 2013<br><br>Briones-Vozmediano et al. 2013<br><br>Hayes et al. 2010<br><br>Hayes et al. 2010	p.234<br><br>p.21<br><br>p.21<br><br>p.388<br><br>p.387
Strained patient-doctor relationships	Patient	"My GP was incensed that I had brought my husband and kicked us out of his office."	Skop, 2015	p.160
	GP	By referring the patient to a rheumatologist the GPs could avoid the difficulties of managing a patient-doctor interaction which escaped their comprehension."<br><br>"If I feel that a patient dislikes me, if I cannot do anything for her and if her judgement does not tell her to go to another doctor, I might behave in a way that makes her abandon me."<br><br>"And then she came once and said to me 'you've treated me very badly, the other day you took no notice of me'. And this feeling that they always think you don't take any notice of them, right?"	Hellstrom et al. 1998<br><br>Hellstrom et al. 1998<br><br>Briones-Vozmediano et al. 2013	p.235<br><br>p.234<br><br>p.22
Paternalistic model	Patient	"My GP was incensed that I had brought my husband and kicked us out of his office."	Skop, 2015	p.160
	GP	"Physicians often described the therapeutic relationship as unidirectional, with patients expected to follow physicians instructions"<br><br>"Define the limits – that's so important, I like that term. That we can't cure them, that we don't fall in with them, that they don't direct [..] what types of pain pills they need or what type of treatment."	Hayes et al. 2010<br><br>Hayes et al. 2010	p.389<br><br>p.389
No one wants to look at it	GP	"I think that the bottom line is no one really wants to look at it."<br><br>"It's a lot of work, it's very unrewarding, you don't really see any concrete results. It's always like a constant decline, so it becomes very unsatisfying as a result".<br><br>"The interviewees, particularly the GPs, began their descriptions with a sigh."	Hayes et al. 2010<br><br>Hayes et al. 2010<br><br>Hellstrom et al., 1998	p.388<br><br>p.389<br><br>p.235
