# Supplementary figures and images for: Patient and primary care practitioners’ perspectives on consultations for fibromyalgia: a qualitative evidence synthesis
Source: Prim Health Care Res Dev. 2023 Sep 26;24:e58. doi: 10.1017/S1463423623000506 (PMC10540196; doi:10.1017/S1463423623000506)

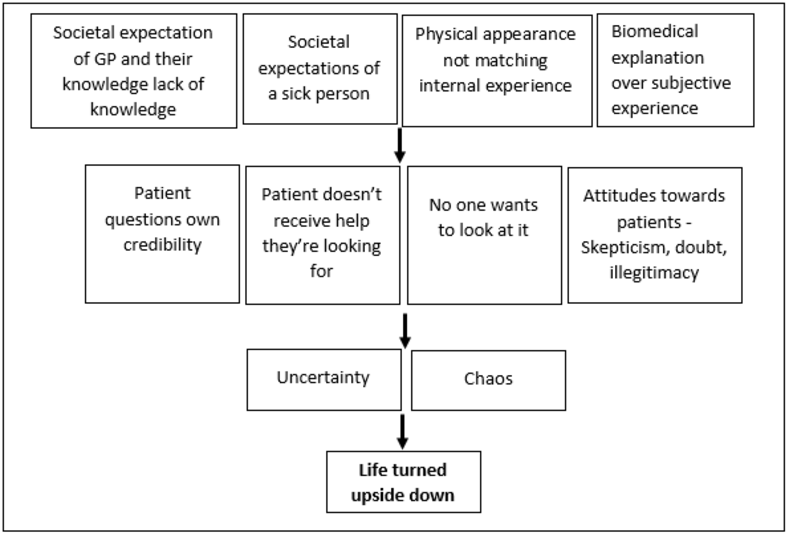

Supplement: Supplementary file 1 [file phcsup.zip › S1463423623000506sup002.tif]

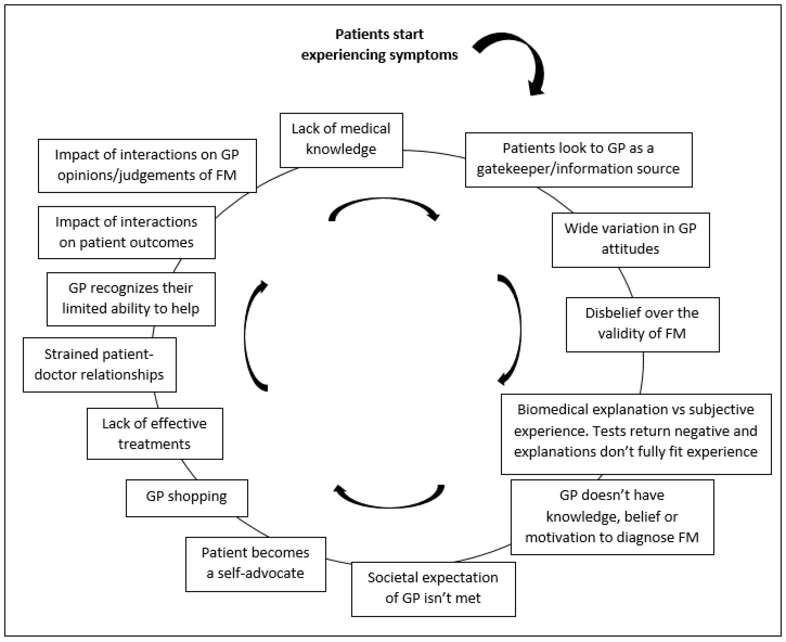

Supplement: Supplementary file 1 [file phcsup.zip › S1463423623000506sup003.tif]

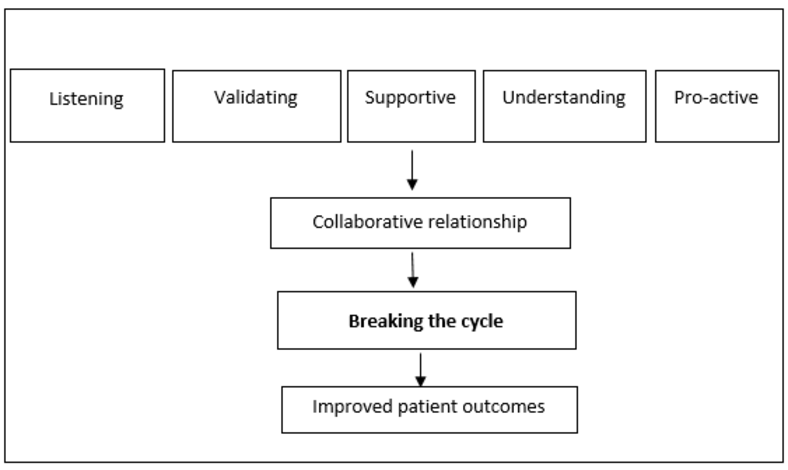

Supplement: Supplementary file 1 [file phcsup.zip › S1463423623000506sup004.tif]
